# Pakistan National Guidelines for Pediatric High-Grade Gliomas

**DOI:** 10.12669/pjms.39.5.6300

**Published:** 2023

**Authors:** Farrah Bashir, Bilal Mazhar Qureshi, Khurram Minhas, Uri Tabori, Eric Bouffet, Cynthia Hawkins, Ather Enam, Naureen Mushtaq

**Affiliations:** 1 Dr. Farrah Bashir, FCPS Aga Khan University Hospital, Karachi, Pakistan; 2 Dr. Bilal Mazhar Qureshi, FCPS Aga Khan University Hospital, Karachi, Pakistan; 3 Dr. Khurram Minhas, FCPS Aga Khan University Hospital, Karachi, Pakistan; 4 Dr. Uri Tabori, MD, PHD The Hospital for Sick Children (SickKids), Toronto, Canada; 5 Dr. Eric Bouffet, MD The Hospital for Sick Children (SickKids), Toronto, Canada; 6 Dr. Cynthia Hawkins, MD The Hospital for Sick Children (SickKids), Toronto, Canada; 7 Dr. Ather Enam, MD Aga Khan University Hospital, Karachi, Pakistan; 8 Dr. Naureen Mushtaq, FCPS Aga Khan University Hospital, Karachi, Pakistan

**Keywords:** Central Nervous System Tumor, Mismatch Repair Genes PMS2, Temozolomide, Germline Mutation, Guidelines

## Abstract

Pediatric high-grade glioma (pHGG) is highly malignant central nervous system tumor and constitute 10% of the pediatric gliomas. Effective treatment needs a functioning multi-disciplinary team including pediatric neuro oncologist, neurosurgeon, neuroradiologist, neuropathologist and radiation oncologist. Despite surgical resection, radiotherapy and chemotherapy, most HGG will recur resulting in early death. A significant proportion of HGG occurs in context of cancer predisposition syndromes like Constitutional Mismatch Repair Deficiency (CMMRD) also known as Biallelic Mismatch Repair Deficiency (bMMRD) characterized by high mutational burden. The incidence of HGG with CMMRD is one per million patients. bMMRD is caused by homozygous germline mutations in one of the four Mis Match Repair (MMR) genes (PMS2, MLH1, MSH2, and MSH6). The use of TMZ is now avoided in CMMRD related HGG due to its limited response and known ability to increase the accumulation of somatic mutations in these patients, increasing the risk of secondary tumors. HGG should be managed under the care of multidisciplinary team to receive optimum treatment. This is particularly important for low middle-income countries (LMIC) with limited resources like Pakistan.

## INTRODUCTION

Children with high grade gliomas (HGG) have a poor prognosis despite the use of multimodal therapy including surgery, radiation and chemotherapy.^1^-^3^ There is scarcity of data regarding prevalence and treatment options for pediatric HGG in low-and middle-income countries (LMIC). Currently radiation with adjuvant and concurrent temozolomide (TMZ) is widely used based on adult data showing improvement in outcome compared with radiation alone.^4^ The Children’s Cancer Group (CCG)-943 trial compared patients treated with radiotherapy alone (standard arm) versus radiotherapy plus lomustine, prednisone, and vincristine (PCV) chemotherapy (experimental arm).^5^ This trial showed that patients in the experimental arm had significant survival advantage (five year EFS 46% versus 18%). In the ACNS0126 trial, patients received concomitant TMZ with radiotherapy followed by 10 courses of adjuvant TMZ.^2^ The results of this trial were disappointing, as TMZ did not seem to result in an improved outcome compared with the therapy provided in the previous CCG-945 trial.

In the recently published phase two study from COG (ACNS0423), following maximal surgical resection, newly diagnosed children with non-metastatic HGG underwent radiotherapy with concurrent TMZ. Adjuvant chemotherapy consisted of up to six cycles of CCNU 90 mg/m2 on day one and TMZ 160 mg/m2/day for five days every six weeks. The one year event free survival was 0.49 (95% CI: 0.039, 0.58) and 3-year overall survival was 0.28 (0.20, 0.37).^6^

Mismatch repair deficiency (MMRD) is caused by mutations or other alterations in one of the four MMR genes (PMS2, MLH1, MSH2, and MSH6). MMRD results in HGG characterized by lack of MMR gene expression by immunohistochemistry, hypermutation and lack of response to temozolomide. MMRD HGG can be caused by previous therapy, somatic mutations in MMR genes or due to germline mutations. Germline mutations in the MMR genes are termed Lynch syndrome in case of heterozygous mutations or CMMRD in case of germline homozygous mutations in the genes.

Constitutional Mismatch Repair Deficiency (CMMRD) (also known as Biallelic Mismatch Repair Deficiency: bMMRD) is an autosomal recessive, highly penetrant cancer predisposition that presents in infancy or young adulthood at an incidence of approximately one per million patients.^5^ bMMRD is caused by homozygous germline mutations in one of the four MMR genes (PMS2, MLH1, MSH2, and MSH6). A 100% of biallelic mutation carriers develop a broad spectrum of early onset tumors, typically within the first two decades of life. High grade gliomas (HGG), characterized by a high mutational burden, are the most common CNS tumors associated with bMMRD and are the major cause of death in these children.^6^-^8^ Despite primary management, which consists of surgical resection followed by radiation therapy and chemotherapy, most HGGs will recur, resulting in rapid death.^8^

Several studies in experimental models indicate that if the mismatch recognition mechanism is not functional, cells become resistant to the killing effects of alkylating agents.^9^ It has been demonstrated that inactivating somatic mutations of the mismatch repair gene MSH6 confers resistance to alkylating agents in gliomas in vivo and fosters a hyper-mutational process in resistant clones because of continued exposure to alkylating agents in the presence of defective mismatch repair.^10^ This is likely to facilitate rapid evolution of clones with growth advantage and contribute to progression.^11^

The loss of MSH6 expression occurs in a significant subset of post-XRT+TMZ recurrent glioblastomas and is associated with the progressive growth of these tumors while they are under TMZ treatment.^11^ The use of TMZ is now avoided in CMMRD related HGG due to its limited response and known ability to increase the accumulation of somatic mutations in these patients, increasing the risk of secondary tumors. We will therefore adopt the backbone of radiotherapy with adjuvant lomustine as per ACNS0423, with the omission of temozolomide as our standard of care for children with MMRD and HGG.

With the help of My Child Matters (MCM) grant, we, as a representative pediatric neuro-oncology team, have developed these guidelines as a standard treatment for children with pediatric HGG in Pakistan. These guidelines will help healthcare professionals to manage HGG in resource limited settings.

## METHODS

A multi-disciplinary writing group was organized which included professionals with experience in managing HGG. This group included pediatric neuro oncologist, neurosurgeons, neuroradiologist, histopathologist and radiation oncologist across Pakistan. These guidelines were reviewed and endorsed by Pediatric Neuro Oncologist at Sick Kids Hospital, Toronto.

### Place of treatment:

Children with high grade glioma should be treated in a hospital that has both the facilities to diagnose and treat them. Special consideration should be given to referral of these children to the Centre with more expertise. This will lead to better survival and outcome of patients. These guidelines include importance of multidisciplinary team. The treating center should have neurosurgical expertise as patient’s outcome is directly related to the maximum resection of tumor. Expert histopathological review along with neuro radiology is of utmost importance. Radiation oncology plays an integral part in imparting treatment. The primary pediatric oncologist should properly guide patients to the centers with appropriate radiation oncology facility. The hospital should be well equipped in giving chemotherapy**.**

It is our view that all HGG cases should be discussed in tumor board meetings regularly with more experienced colleagues. In this way capacity building occurs in Pakistan, and risk can be assessed and discussed.

## DIAGNOSIS:

### Presentation:

Because of the rapid growth of tumor, the duration of symptoms is short. Symptoms related to increased intracranial pressure include persistent headaches, early morning vomiting and diplopia. Focal deficits can occur depending upon tumor location. Seizures are seen less often with HGG but if tumor involves temporal lobe, then seizures can occur.

Patients with CMMRD can have café-au-lait spots. Many factors contribute to delay in diagnosis and aggressive disease at presentation. Some of these are traveling distance from treatment facility, financial constraints and knowledge proper referral pathway.

### Radiology:

Radiological studies are the keystone in diagnosing brain tumor. The most common modality used in low middle-income countries for diagnosing is CT scan both plain and post contrast views. Th investigation of choice is MRI Brain with contrast. It should be done in all patients suspected of having HGG, who are referred prior to surgery. If surgery has been performed in an outside hospital, then all pre-surgery scans should be collected and reviewed by the treating doctor. All patients should undergo brain MR imaging at least at 0.5T.

### Following sequences should be obtained:

axial and coronal T2 FSE (TR/TE, 2700/100 ms), axial or Coronol FLAIR (TR/TE, 9000/120 ms; TI, 2200 ms), pre contrast T1 spin-echo and contrast-enhanced T1 spoiled gradient-recalled echo (TR/TE, 8/3 ms; 1-mm section thickness, 0 skip), followed by 2 planes of contrast-enhanced T1 spin-echo (TR/TE, 600–700/20 ms; 5-mm section thickness, 0.5 skip). All, patients should undergo DWI; b-value of 1000 s/mm2; 3 directions; 4-mm thickness, 0 skip) SWI/GRE/T2* is optional.

### All reports should comment on:

1.Tumor location, 2. Enhancement pattern 3. Cysts/cavities, 4. Hemorrhage/ mineralization, 5. Intracranial or leptomeningeal seeding, 6. Tumor margin, 7. Necrosis as suggested by ring-enhancement

“Tumor margin” should be characterized as ill-defined if >50% of the margin could not be distinguished from the surrounding cerebellar parenchyma based on all imaging sequences.

“Enhancement pattern” should be defined as minimal/none if <10% was estimated to enhance, solid if >90% of the tumor volume was estimated to enhance, and heterogeneous if varying degrees of enhancement were seen in 10%–90% of the tumor volume based on radiologist’s visual assessments. Low signal on 2D gradient recalled echo or bright on T1W should be used to detect hemorrhage/mineralization.

**Fig.1:**
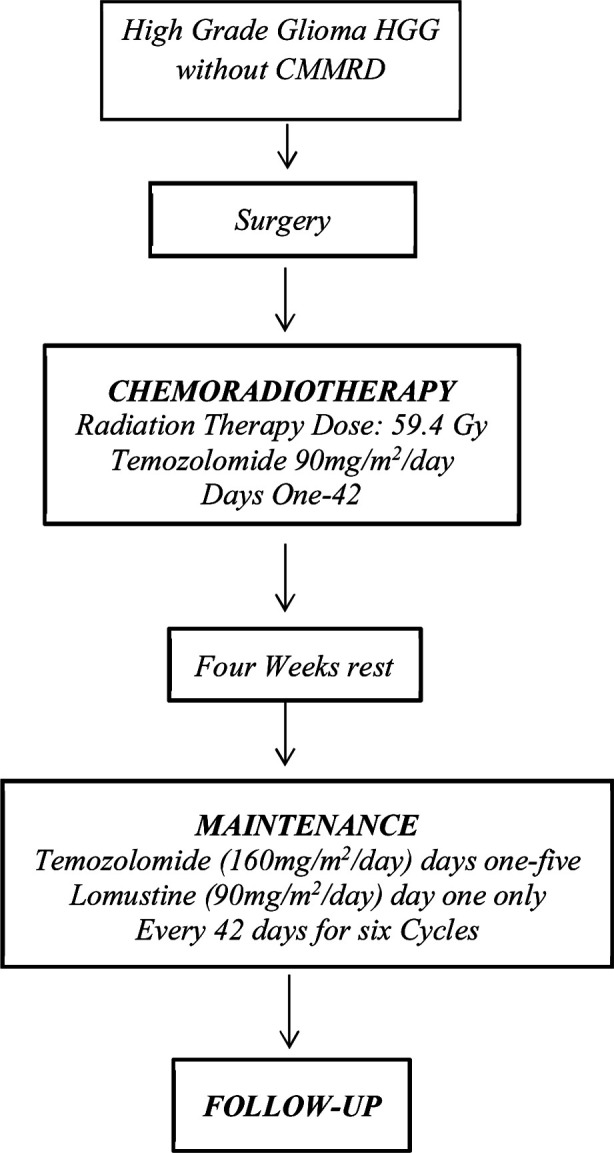
Flow chart One.

**Fig.2:**
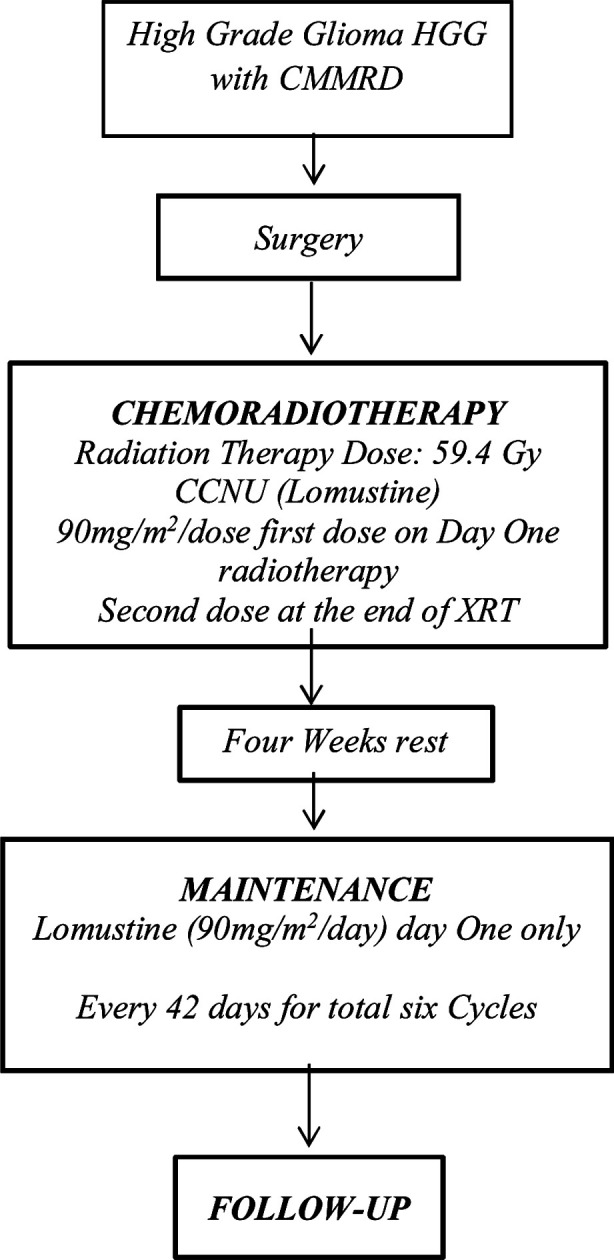
Flow chart Two.

Tumour size should be given in three dimensions and try best to give volume. Formula for tumour volume is:

Tumor volume = length x width2/2,

Where length represents the largest **tumor** diameter and width represents the perpendicular tumor diameter.

Measurements should be taken on postcontrast or T2W/FLAIR.

### Histopathology:

Pediatric high-grade gliomas (pHGG) constitute 10% of the pediatric gliomas. It is a heterogenous category of aggressive tumors with poor prognosis accounting for over 40% of deaths due to Primary CNS tumours^12^. Recent advances in Molecular profiling have led to revision in classification of these tumors. The recent WHO classification, 5^th^ Edition^13^, classifies these as a) Diffuse midline glioma, H3 K27-altered, WHO grade 4 b) Diffuse hemispheric glioma, H3 G34-mutant, WHO grade 4 c) Diffuse pediatric-type high-grade glioma, H3-wildtype and IDH-wildtype, WHO grade 3 or 4 d) Infant-type hemispheric glioma, WHO grade 4. For accurate diagnoses, specific immunohistochemical markers and molecular testing is required. However, in low- and middle-income countries (LMIC) where molecular studies are yet not available, immunohistochemical stains (H3K27M, H3K27me3, H3G34R/V, IDH-1, R132H, ATRX, p53 olig-two and BRAF V600E) can be used in classifying high grade glioma according to the recent WHO classification for clinical management purpose.

In addition, to screen for CMMRD in cases of pHGG, A panel of four immunohistochemical stains (MLH-1, MSH2, MSH6 and PMS2) are required. If there is loss of staining of one or more markers then genetic workup is recommended to identify CMMRD patients.^7^,^8^ It is suggested that screening for CMMRD be performed in all cases of pHGGs.

### Guidelines for Replication Repair Deficiency screening in CAYA gliomas:

Although the prevalence of RRD (replication repair deficiency) in high grade gliomas (HGG, WHO grade III, IV gliomas) of children and younger adults (CAYA, ages zero-40 years) is unknown, the potential benefit from detecting this mechanism is substantial. RRD affects response to chemotherapy such as temozolomide^12^ and results in potential significant survival benefit using immune checkpoint inhibition.^8^ Furthermore, as significant numbers of the RRD-HGG will be caused by germline mutations in either the mismatch repair genes or DNA polymerase, accurate diagnosis will affect the patient and family members.^14^

It is therefore suggested that all CAYA HGG will be screened initially by immunohistochemistry for the mismatch repair proteins (PMS2, MSH6, MSH2, MLH1) to evaluate for loss of staining in both tumor and in case of CMMRD, adjacent normal tissue. Although only 85-90% sensitive, this is good and cheap tool for initial screen.^15^,^16^ RRD is highly recommended in clinical scenarios with significant past or family history of cancers, especially gastrointestinal, genitourinary, CNS and hematopoietic cancers^17^, consanguinity and Phenotypic manifestations such as café au lait spots, pilomatrixomas, or multiple developmental venous anomalies in the brain.^18^,^19^ RRD is highly recommended in cases with molecular pathology including anaplastic PXA, IDH1 mutant gliomas in CAYA, lacking BRAF,RET,ALK fusions, RTK1 HGG by methylation arrays^20^ and all gliomas with high tumor mutation burden (>five) by sequencing.^12^,^14^ The most sensitive and specific tool to assess RRD in HGG is based on whole genome MS-sigs. This robust tool can be done from paraffin tissue and can detect both MMR and polymerase dysfunction.

## TREATMENT GUIDELINES:

Treatment of HGG included maximum safe resection followed by radiation therapy and chemotherapy. The extent of tumor resection is very important in providing survival benefit.

### Neurosurgery:

Surgical resection is the cornerstone of management of HGG with the goals of establishing diagnosis, alleviating symptoms due to elevated intracranial pressure and mass effect, improving outcomes, and delivering local therapies.

There are no standard neurosurgical procedures for high-grade astrocytomas. The extent of surgical resection will depend upon the tumor location within the brain and its vascularity. Patients potentially eligible for this study will undergo a neurosurgical procedure in which the diagnosis will be pathologically confirmed. Gross total resection should be performed whenever possible; if not feasible, an attempt to do maximum safe resection without jeopardizing the patient should be made. If even a subtotal or partial resection is considered hazardous to the patient, then a biopsy procedure must be performed to make a pathologic diagnosis. No patient will be eligible for this study without a pathologic diagnosis and sufficient specimens for the required biologic studies. Corticosteroids can be administered preoperatively to reduce tumor-associated edema unless primary cerebral lymphoma or inflammatory lesions are suspected, Table-I.

**Table-I:** Definitions of extent of resection.

Extent of Resection	Definition
Biopsy only	Open or closed (e.g., needle) surgical removal of tissue for the sole purpose of making a pathologic diagnosis. If tumor removal is <10% of the total tumor mass, this will be considered a biopsy only.
Partial resection	Surgical removal of >10%, but <50% of the tumor mass.
Subtotal Resection	Surgical removal of ≥50%, but <90%, of the tumor mass.
Extensive Subtotal Resection	Resection of ≥90% of the tumor mass, but residual disease apparent on inspection.
Gross Total Resection	Resection of all visible tumor.

All patients must have confirmation of the neurosurgical opinion of the extent of resection by a postoperative enhanced MRI scan. Immediate postop scan should be performed in 24-48 hours. Postoperative complications include unexpected neurological deficits, CSF leak, postoperative seizures, death, infection, and the need for revision surgery. The patient should be kept in the ICU if deemed necessary due to unstable vital signs or poor neurological status and transferring out of ICU should be considered after stabilization. Urinary catheters should be removed on postoperative day one or as soon as possible. In stable patients, the diet can be progressed after surgery as tolerated. Postoperative electrolyte and hematological studies should be ordered and if given, anticonvulsant levels should also be checked. Patients must be discharged as soon as possible as early discharge expedites adjuvant chemo-radiation therapies and recovery time.

### Chemotherapy:

All patients must begin therapy within 31 days of their surgical resection. There are two treatment protocols for HGG patients. If the patient is positive for CMMRD then temozolomide is omitted. Chemo-radiotherapy phase without CMMRD will receive radiotherapy as 54 Gy over six weeks followed by a boost of an additional 5.4 Gy for a total of 59.4 Gy. During radiotherapy, patients will start TMZ within five days of starting radiation, daily for 42 days regardless of end date of radiation and accounting for interruptions. TMZ should be given orally in morning with antiemetics (given 30 minutes before each dose)^21^. TMZ dose during radiotherapy is 90mg/m^2^/day if BSA >0.5m^2^ (3mg/kg/day if BSA≤ 0.5m^2^) for 42 days (round the dose to nearest five mg). (Flow chart One)

Prophylactic Trimethoprim/Sulfamethoxazole (septran) should not be utilized as PCP prophylaxis during chemo-radiotherapy and instead monthly IV pentamidine is given. Trimethoprim/Sulfamethoxazole may be used as PCP prophylaxis during maintenance chemotherapy. Maintenance chemotherapy starts four weeks post completion of radiotherapy. It consists of six cycles of combination TMZ and Lomustine, cycles repeated every six weeks provided ANC ≥1000/μL, platelet Count ≥ 100,000/μL (transfusion independent), serum creatinine ≤1.5 x normal for age and total bilirubin ≤ 1.5 x normal for age, and ALT and AST ≤ 2.5 x normal for age. Five days TMZ (day one-five) and one dose of Lomustine (on day one) followed by 36 days of rest is considered one treatment cycle. TMZ dose during maintenance cycles is 160mg/m2/day if BSA>0.5m^2^ (4.6 mg/kg/day if BSA≤ 0.5m^2^), round the dose to the nearest five mg. Lomustine dose during maintenance cycles is 90mg/m^2^/day if BSA >0.5m^2^ (2.5mg/kg/day if BSA≤0.5m^2^), round to the nearest 10mg.

In patients of HGG with CMMRD, Radiation therapy should be started within 31 days of surgery. Patients will begin Lomustine on Day one of radiotherapy. A second dose of Lomustine will be given at the end of radiotherapy.^6^ Patients will begin Maintenance therapy with Lomustine on day one of each cycle, if patient meets criteria as previously outlined. Each cycle will last for 42 days. Patient will receive six cycles of Lomustine with a total of eight doses of Lomustine. (Flow chart Two)

If patient is unable to swallow capsule, the entire capsule contents of the required lomustine capsules may be mixed with food such as applesauce, yogurt, or jam. Lomustine should be given at bedtime on an empty stomach (one hour before or two hours after food). A vomited dose should not be repeated if it occurs more than 30-45 mins after the dose. Oral etoposide is prepared by properly mixing injectable etoposide with 0.9% sodium chloride solution and can be kept at room temperature for 22 days. Solution can be further diluted immediately prior to administration in apple juice, orange juice or lemonade (NOT grapefruit juice) and should be kept away from sunlight.^22^,^23^

### Radiation:

Radiation Therapy is to start within 31 days of the definitive surgical procedure. Primary brain malignant gliomas will receive a total dose of between 54.0 and 59.4 Gy in 30-33 fractions over six-seven weeks. The total dose will be 54.0 Gy if a gross total resection (GTR) has been performed. If the tumor has not been completely resected, the residual disease will be boosted to a total dose of 59.4 Gy. Primary spinal cord malignant gliomas will receive a total dose of between 50.4-54 Gy in 28-30 fractions over five - six weeks, regardless of the extent of tumor resection.

No treatment breaks in the radiation therapy component of this combined modality treatment are anticipated. Skin reactions should be treated supportively. Low blood counts are generally related to systemic therapy and are not caused or worsened by local field brain RT.

### Recurrent High-grade glioma:

Six cycles of oral Etoposide can be given to patients with recurrent high grade glioma patients. It is given for 21 days with a dose of 50 mg/m^2^. If the extent of resection (EOR) is >80% and site of recurrence is distant then there are chances of better survival with a median overall survival of 1.9 years.^24^ Decision for re-irradiation be taken for highly selected cases only through a site-specific pediatric neuro oncology multidisciplinary tumor board meeting with the presence of expert pediatric neurosurgeon, pediatric neuro-oncologist, pediatric neuro-radiologist and dedicated site-specific radiation oncologist. Previous radiotherapy plan with CT data of dosimetry and iso dose curved should be available for previous radiation treatment plans.

Operative factors associated with better survival include extent of resection >80% (EOR) and distant site of recurrence

### Supportive care:

Supportive care is provided in the form of antiemetics, antibiotics and blood products like platelets and red blood cells. Blood products will be irradiated to prevent graft-versus-host disease. Filters should be used to prevent WBC sensitization.

### Follow up:

MRI Brain with contrast (MRI Spine when indicated) should be done at start of treatment, before first cycle of chemotherapy, at 3^rd^ cycle and at 6^th^ cycle. Then at every four months for first year, six monthly for next two years and yearly till 5^th^ year of surveillance.

### Conclusion:

In LMIC like Pakistan pediatric HGG should be treated with dedicated pediatric neuro-oncology teams to achieve better outcomes.^25^ Studies show rate of consanguinity approximately 60% in Pakistan^26^ and in another study first cousin marriages account for more than 50% cases.^27^ It is assumed that due to high rate of consanguinity, there are higher number of CMMRD patients with HGG, so more focused studies should be done in this part of the world.

**Table:** 

Dr. Gohar Javed	Aga Khan University Hospital Karachi, Pakistan
Dr. M. Waqas Saeed Baqai	Aga Khan University Hospital Karachi, Pakistan
Dr. Shahzad Shamim	Aga Khan University Hospital Karachi, Pakistan
Dr. Ehsan Bari	Aga Khan University Hospital Karachi, Pakistan
Dr. Anila Darbar	Aga Khan University Hospital Karachi, Pakistan
Dr. Fatima Mubarak	Aga Khan University Hospital Karachi, Pakistan
Dr. Syed Ahmer Hamid	Indus Hospital and Health Network Karachi, Pakistan
Dr. Ata ur Rehman Maaz	Sidra Medicine Hospital, Doha, Qatar
Dr. Uzma Imam	National Institute of Child Health Karachi, Pakistan
Dr. Nuzhat Yasmeen	Pakistan Institute of Medical Sciences Islamabad, Pakistan
Dr. Zulfiqar Ali Rana	Children Hospital, Multan, Pakistan
Dr. Shazia Riaz	Children Hospital Lahore, Pakistan
Dr. Alia Ahmed	Children Hospital Lahore, Pakistan
Dr. Najma Shaheen	Shaukat Khanam Cancer Memorial Hospital Lahore, Pakistan
Dr. Tariq Ghafoor	Combined Military Hospital, Rawalpindi, Pakistan

### Author’s Contribution:

**FB:** Handled the compilation and editing the article.

**BMQ:** Prepared the radiation therapy guidelines.

**KM, CH and UT:** Helped in the histopathology guidelines.

**EB:** Reviewed the guidelines completely and his input was incorporated as well.

**AE:** Prepared Neurosurgical guidelines.

**NM:** Conceived, designed, and drafted the chemotherapy guidelines and whole treatment schema.
